# SARS-CoV-2 RNA Shedding in Semen and Oligozoospermia of Patient with Severe Coronavirus Disease 11 Weeks after Infection

**DOI:** 10.3201/eid2801.211521

**Published:** 2022-01

**Authors:** Lawrence J. Purpura, Joseph Alukal, Alexander M. Chong, Lihong Liu, Anyelina Cantos, Jayesh Shah, Nicola Medrano, Jennifer Y. Chang, Moriya Tsuji, Hiroshi Mohri, Anne Catrin Uhlemann, David Ho, Michael T. Yin

**Affiliations:** Columbia Mailman School of Public Health, New York, New York, USA (L.J. Purpura);; Columbia University Irving Medical Center, New York (L.J. Purpura, J. Alukal, A.M. Chong, L. Liu, A. Cantos, J. Shah, N. Medrano, J.Y. Chang, M. Tsuji, H. Mohri, A.C. Uhlemann, D. Ho, M.T. Yin)

**Keywords:** coronavirus disease, COVID-19, severe acute respiratory syndrome coronavirus 2, SARS-CoV-2, viruses, shedding, respiratory infections, semen, zoonoses

## Abstract

We report severe acute respiratory syndrome coronavirus 2 in semen by using quantitative reverse transcription PCR during the late convalescent phase. Virus was associated with adequate humoral and cell-mediated responses, suggesting possible seeding of the immune-privileged testes. We provide longitudinal semen quality data for 6 other men, including 3 who had oligozoospermia.

As of July 2021, >180 million persons worldwide were infected by severe acute respiratory syndrome coronavirus 2 (SARS-CoV-2) and remain in the convalescent phase (*1*). Long-term implications for male fertility and potential sexual transmission remain uncertain. However, other emerging pathogens, such as Ebola and Zika viruses, have been shown to undergo sexual transmission (*2*,*3*).

Angiotensin-converting enzyme-2 receptors, abundant in the testis (*4*), are binding sites for SARS-CoV-2, and autopsy reports have demonstrated viral invasion of the testis (*5*). However, detection of SARS-CoV-2 RNA in semen was not reported from 6 cohort studies that included 213 men (*6*), although detection has been reported in 2 studies during the acute phase (*7*,*8*) and in 1 study during early convalescence (*9*). We present findings of semen analysis from a prospective coronavirus disease (COVID-19) cohort study. 

## The Study

A 34-year-old man, who had a history of childhood asthma, was hospitalized for severe coronavirus disease (COVID-19) pneumonia during March 2020. At admission, he had symptoms for 1 week and was positive for SARS-CoV-2 RNA by nasopharyngeal swab specimen reverse transcription PCR (RT-PCR). Chest radiograph showed bilateral interstitial opacities. He was given supplementary oxygen by nasal cannula and completed a 5-day course of hydroxychloroquine but was also given mechanical ventilation on day 10 for respiratory decompensation. His hospital course was complicated by renal failure requiring continuous venovenous hemofiltration. He was eventually extubated on illness day 15 but remained admitted until day 27, when he no longer required supplemental oxygen. Despite his respiratory recovery, he required outpatient dialysis until day 51. At the time of study enrollment (day 72), he had returned to his previous state of health.

Participants were recruited for this prospective cohort study from inpatient and outpatient settings in New York, New York, beginning in March 2020. The study was approved by the institutional review board at Columbia University Irving Medical Center and is registered at Clinicaltrials.gov (NCT04448145). Eligible participants had laboratory confirmation of COVID-19 based on SARS-CoV-2 RT-PCR or serologic testing. Participants completed surveys describing their demographics, underlying conditions, and COVID-19 clinical course. Clinical samples collected at each visit included plasma, peripheral blood mononuclear cells, nasopharyngeal swab specimens, saliva samples, stool/rectal swab specimens, and semen.

We collected and assessed semen per World Health Organization guidelines (*10*). We instructed participants to clean their hands without spermotoxic lubricants before providing a sample into a sterile container. Samples were frozen after collection. Sperm count was reported in millions per milliliter, and sperm mho provided semen specimens were Hispanic, 2 Black, and 3 Caucasian; 2 had a previous diagnoses of HIV infection, and 2 had body mass index >30 kg/mg^2^. One participant had had a successful vasectomy and was included in the study to evaluate viral carriage in nontestes accessory organs, such as the prostate gland.

Of the 107 patients enrolled in the cohort study, 7 provided semen specimens (Table 1; Appendix). The mean age of participants was 38.7 (range 32–56) years. Two of the 7 who provided semen specimens were Hispanic, 2 Black, and 3 Caucasian; 2 had a previous diagnoses of HIV infection, and 2 had body mass index >30 kg/mg^2^. One participant had had a successful vasectomy and was included in the study to evaluate viral carriage in nontestes accessory organs, such as the prostate gland.

**Table 1:** SARS-CoV-2 RT-PCR and semen analysis for 7 participants enrolled in a longitudinal prospective coronavirus disease cohort study, New York, USA*

**Participant ID**	**Days to specimen collection**	**Semen RT-PCR result **	**Sperm count, million sperm/mL semen**	**Semen motility, %**
1	81	Positive	<1	0
	101	Negative	16	0
	170	Negative	72	0
2	38	Negative	3	0
3	48	Negative	60	0
	65	Negative	82	40
4	61	Negative	158	50
	68	Negative	44	30
	74	Negative	30	20
5	93	Negative	5	10
	102	Negative	34	0
	115	Negative	30	0
	335	NT	16	0
6	37	Negative	12	50
7	84	Negative	Vasectomy	NT
	94	Negative	Vasectomy	NT
	101	Negative	Vasectomy	NT

*ID, identification; NT, not tested; RT-PCR, reverse transcription PCR; SARS-CoV-2, severe acute respiratory syndrome coronavirus 2.

A total of 17 semen specimens were collected from 7 participants (Table 1) and underwent quantitative RT-PCR (qRT-PCR) testing and semen analysis. We assessed cycle threshold (C_t_) values by using the ZymoBIOMICS DNA/RNA Extraction Kit (Zymoresearch, https://www.zymoresearch.com), the Taqman 4× One-Step Master Mix (Zymoresearch), and SARS-CoV-2 IDT primer/probe sets (Integrated DNA Technologies, https://www.idtdna.com). One sample obtained from participant 1 on day 81 from symptom onset was qRT-PCR positive and had a C_t_ value of 34.79. Virus isolation was unsuccessful (Appendix). Subsequent semen samples from the participant at days 101 and 169 were negative, as were his saliva, stool, and plasma samples (Table 2). All semen samples from the other 6 men were negative by qRT-PCR.

**Table 2:** RT-PCR and serologic test results for participant 1 who was infected with SARS-CoV-2 in longitudinal prospective coronavirus disease cohort study, New York, USA*

**Days after symptom onset**	**Sample**	**PCR result, C_t_**	**Binding antibody EC_50_, NP/S**	**Neutralizing antibody IC_50_**	**T cell reactivity against M protein, IFN-γ‒secreting T cells/million PBMCs†**
72	Saliva	Negative			
72	Blood	Negative	1,351/3,728 (modest)	(modest)	
78	Saliva	Negative			
78	Stool	Negative			
78	Blood	Negative	1,756/5,680 (modest)	(modest)	
81	Semen	Positive, 34.79			
81	Blood	NT			>130 (strong)
81	Saliva	Negative			
101	Semen	Negative			
101	Saliva	Negative			
101	Stool	Negative			
169	Saliva	Negative			
169	Stool	Negative			
169	Semen	Negative			

*C_t_, cycle threshold; EC_50_, concentration half of the maximum effect; IC_50_, half-maximal inhibitory concentration; IFN, interferon; NP, nucleocapsid protein; NT, not tested; PBMCs, peripheral blood mononuclear cells; S, spike protein; SARS-CoV 2, severe acute respiratory syndrome coronavirus 2.

Participant 1 had severe oligozoospermia and a sperm concentration of <1 million/mL on day 81, followed by a gradual recovery to 16 million/mL on day 101 and 72 million/mL on day 170. All his samples showed sperm motility of 0%, although samples were previously frozen. In addition, 2 other participants had severe oligozoospermia (<5 million/mL) and 1 had mild oligozoospermia (<15 million/mL). Follow-up samples were available for 5 participants. Early sperm count recovery was observed in 3 participants, but 2 participants had a decrease in sperm count later in convalescence, and 1 participant had a count of 16 million/mL at 11 months (Table 1).

**Figure 1:**
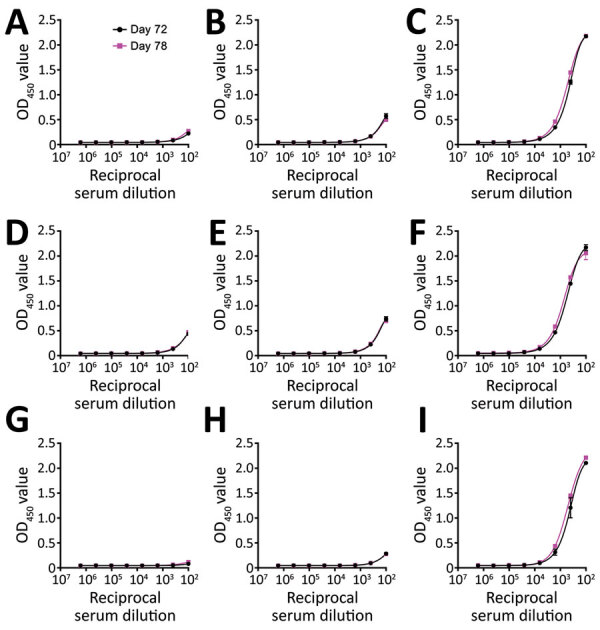
IgA, IgM, and IgG antibody responses against severe acute respiratory syndrome coronavirus 2 (SARS-CoV-2) spike protein, receptor-binding domain (RBD), and nucleocapsid protein (NP) for participant 1 at days 72 and 78 from symptom onset. A) C0087 plasma SARS-CoV-2 spike-specific IgA; B) C0087 plasma SARS-CoV-2 spike-specific IgM; C) C0087 plasma SARS-CoV-2 spike-specific IgG; D) C0087 plasma SARS-CoV-2 spike/RBD‒specific IgA; E) C0087 plasma SARS-CoV-2 spike/RBD‒specific IgM; F) C0087 plasma SARS-CoV-2 spike/RBD‒specific IgG; G) C0087 plasma SARS-CoV-2 NP-specific IgA; H) C0087 plasma SARS-CoV-2 NP-specific IgM; I) C0087 plasma SARS-CoV-2 NP-specific IgG. OD_450_, optical density at 450 nm.

**Figure 2:**
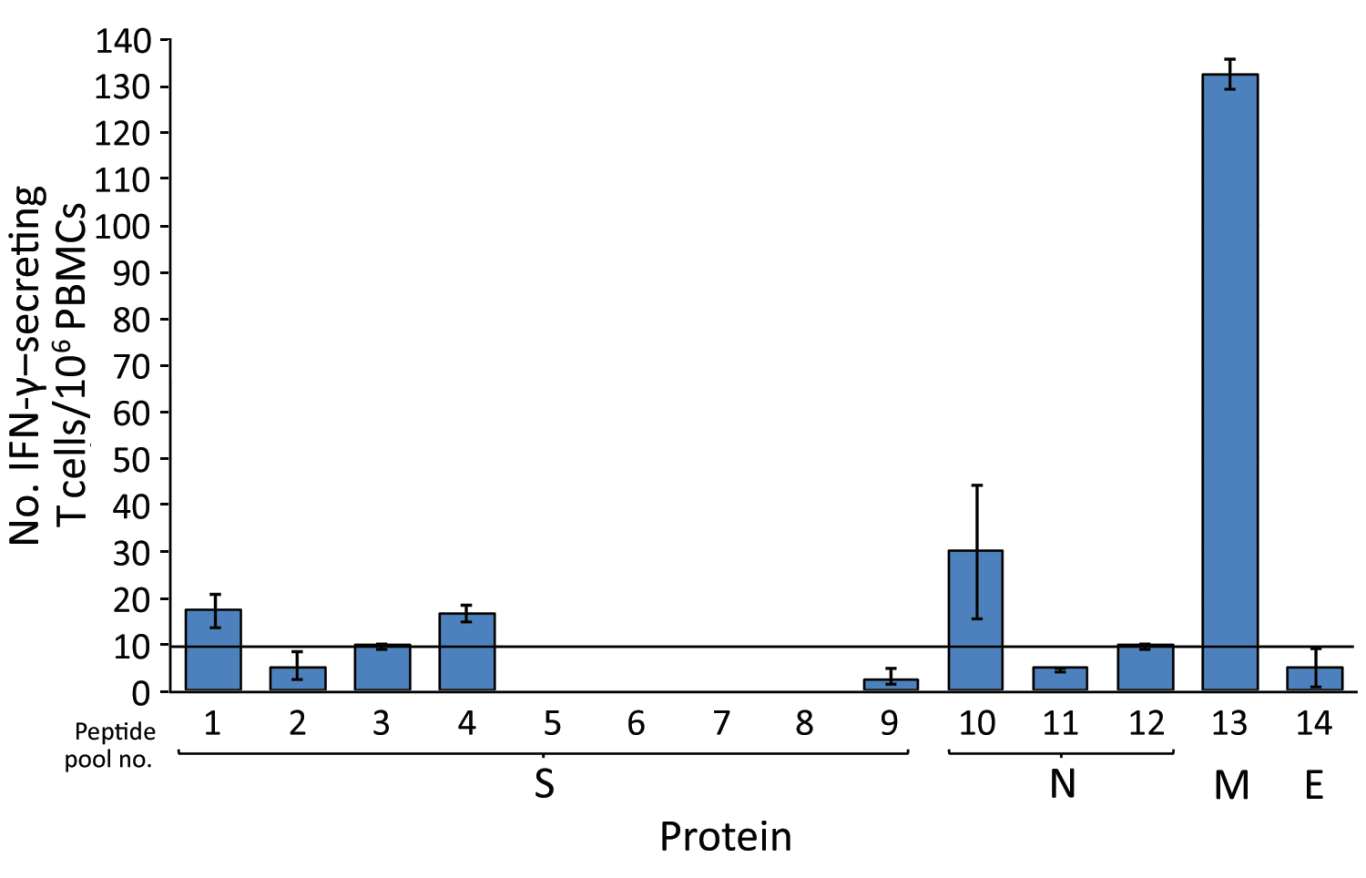
T-cell binding domain responses against membrane, nucleocapsid, spike, and envelope proteins of severe acute respiratory syndrome coronavirus 2, as determined by human IFN-γ ELISpot assay. If the number of spots that correspond to the number of IFN-γ‒secreting T cells/million PBMCs is >10, the response is considered positive. Peptide pool numbers indicate 17-mer overlapping peptides that encompass all 4 proteins. IFN, interferon; PBMCs, peripheral blood mononuclear cells. Error bars indicate mean ± SD.

We assessed humoral and cell-mediated immune responses to evaluate level of immunity against SARS-CoV-2. We used immunoassays (*11*) to quantify IgM, IgG, and IgA binding (half of maximum effect) values against spike trimer and nucleocapsid protein (Figure 1). We performed antibody neutralization assays to measure the neutralization half-maximal inhibitory concentration (Figure 1; Appendix). Half of the maximum effect binding antibody responses and neutralization half-maximal inhibitory concentration against SARS-CoV-2 trimer were modest at days 72 and 78 (Table 2). We used a human interferon-γ ELISpot assay to determine the T-cell response against spike trimer, nucleocapsid, matrix, and envelope proteins at day 81 from symptom onset, which showed strong reactivity against matrix (Figure 2).

## Conclusions

We report detection of SARS-CoV-2 RNA by qRT-PCR in semen and severe oligozoospermia in 1 patient 81 days after onset of severe COVID-19. Compared with reports that showed positive RT-PCR findings (*7*–*9*), we provide more granularity regarding clinical course, longitudinal assessment of sperm count, and host immune response.

Detection of SARS-CoV-2 RNA during the late convalescent phase might be attributed to COVID-19 severity, requiring mechanical ventilation and renal replacement therapy for the participant. This feature might have resulted in an enhanced systemic viremic state with subsequent seeding of accessory organs or the testes, an immune-privileged site, in the setting of generalized inflammation, and disruption of the blood–testis barrier (*12*). This possibility is supported by the participant having adequate humoral and cell-mediated immune responses and associated viral clearance of stool and saliva specimens surrounding the time when SARS-CoV-2 RNA was detected in semen.

In addition, 4 other study participants had oligozoospermia. Sperm count recovery was observed in 2 participants, but the other 2 did not provide longitudinal samples. Numerous reports have suggested a detrimental effect on semen quality after COVID-19 (*9*,*13*,*14*), hypothesized to occur secondary to viral illness and fever causing spermatogenic dysfunction (*15*). Given this transient insult, it is not unexpected that some of these men showed recovery.

One limitation of our study is that the initial semen sample was collected late in the convalescent phase, and the high C_t_ value probably indicates detection of inactive virus without risk for sexual transmission. However, if an acute-phase or early convalescent-phase specimen were collected, the C_t_ value might have been lower. Likewise, semen samples from the other 6 men were also limited to the late convalescence phase and mild acute COVID-19 illnesses, except for 1 participant who required mechanical ventilation, although his status was postvasectomy.

Given the small sample size, we cannot determine the contribution of other known etiologies of oligozoospermia, including obesity and oxygen therapy during hospitalization. In addition, we lacked preinfection semen analysis for comparison, and sperm motility would have been more accurately assessed if performed before freezing. Last, because of inherent difficulty in recruiting for serial semen collection, semen was only collected from a small proportion of participants enrolled in the cohort study. These findings are not generalizable to all male COVID-19 survivors and warrant further research.

In conclusion, SARS-CoV-2 RNA in semen appears to be an extremely rare event, but oligozoospermia has been reported more frequently. Risk factors for viral persistence in the male reproductive tract, longitudinal effects on semen quality, and viral transmission remain to be elucidated, but because of the large number of men in the convalescent phase worldwide, potential effects on reproductive health is not negligible.

Appendix Additional information on SARS-CoV-2 shedding in semen and oligozoospermia in patient with severe coronavirus disease 11 weeks after infection.
