# Supplementary material for: SARS-CoV-2 RNA Shedding in Semen and Oligozoospermia of Patient with Severe Coronavirus Disease 11 Weeks after Infection
Source: Emerg Infect Dis. 2022 Jan;28(1):196–200. doi: 10.3201/eid2801.211521 (PMC8714206; doi:10.3201/eid2801.211521)
Supplement: Appendix — Additional information on SARS-CoV-2 shedding in semen and oligozoospermia in patient with severe coronavirus disease 11 weeks after infection. [file 21-1521-Techapp-s1.pdf]

Article DOI: https://doi.org/10.3201/eid2801.211521

SARS-CoV-2 Shedding in Semen and Oligozoospermia in Patient with Severe Coronavirus Disease 11 Weeks after Infection

Appendix.

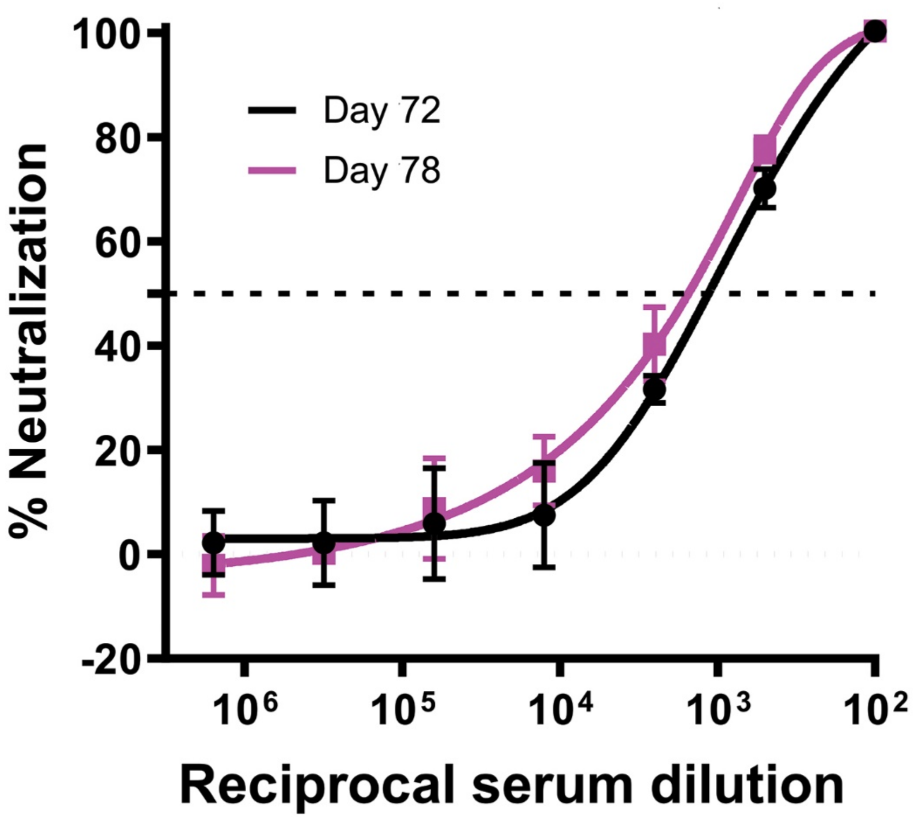

Appendix Figure. Severe acute respiratory syndrome coronavirus 2 neutralization half-maximal inhibitory concentration in plasma at days 72 and 78 postsymptom onset. Dashed line indicates 50% neutralization.
